# Crystal structure of bis­(1,3-di­meth­oxy­imidazolin-2-yl­idene)silver(I) hexa­fluorido­phosphate, N-heterocyclic carbene (NHC) complex

**DOI:** 10.1107/S2056989015023130

**Published:** 2015-12-09

**Authors:** Barbara Rietzler, Gerhard Laus, Volker Kahlenberg, Herwig Schottenberger

**Affiliations:** a University of Innsbruck, Faculty of Chemistry and Pharmacy, Innrain 80, 6020 Innsbruck, Austria; b University of Innsbruck, Institute of Mineralogy and Petrography, Innrain 52, 6020 Innsbruck, Austria

**Keywords:** crystal structure, silver(I), 1,3-di­meth­oxy­imidazolin-2-yl­idene, hexa­fluorido­phosphate salt

## Abstract

The title salt, [Ag(C_5_H_8_N_2_O_2_)_2_]PF_6_, was obtained by deprotonation and metalation of 1,3-di­meth­oxy­imidazolium hexa­fluorido­phosphate using silver(I) oxide in methanol. The C—Ag—C angle in the cation is 178.1 (2)°, and the N—C—N angles are 101.1 (4) and 100.5 (4)°. The meth­oxy groups adopt an *anti* conformation. In the crystal, anions (*A*) are sandwiched between cations (*C*) in a layered arrangement {*C*…*A*…*C*}_*n*_ stacked along [001]. Within a *C*…*A*…*C* layer, the hexafluoridophosphate anions accept several C—H⋯F hydrogen bonds from the cationic complex.

## Related literature   

For synthesis of 1,3-di­meth­oxy­imidazolium hexa­fluorido­phosphate, see: Laus *et al.* (2007 ▸). For related structures, see: Laus *et al.* (2008 ▸, 2010 ▸). For background to *N*-heterocyclic carbene (NHC)–silver complexes, see: Garrison & Youngs (2005 ▸); Lin *et al.* (2009 ▸); Lin & Vasam (2007 ▸); Wang & Lin (1998 ▸). For the nature of C—H⋯F inter­actions, see: D’Oria & Novoa (2008 ▸).
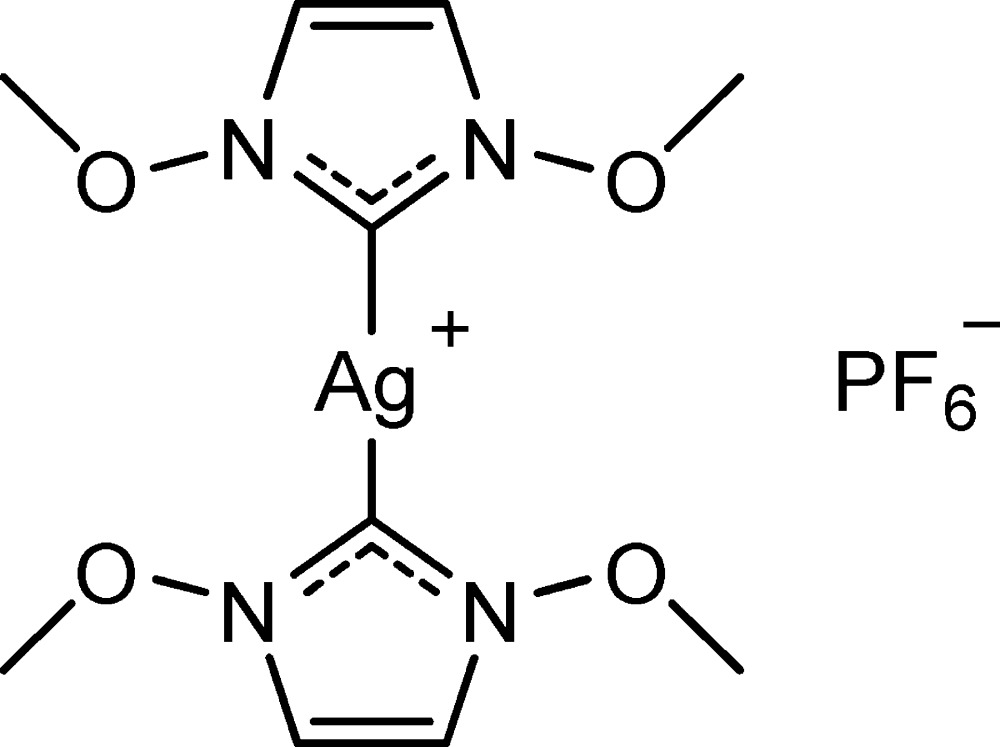


## Experimental   

### Crystal data  

[Ag(C_5_H_8_N_2_O_2_)_2_]PF_6_
*M* *_r_* = 509.11
Triclinic, 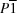

*a* = 7.5254 (7) Å
*b* = 11.7221 (12) Å
*c* = 11.8697 (12) Å
α = 109.481 (9)°
β = 100.698 (8)°
γ = 100.052 (8)°
*V* = 937.84 (17) Å^3^
*Z* = 2
Mo *K*α radiation
μ = 1.24 mm^−1^
*T* = 243 K
0.25 × 0.12 × 0.05 mm

### Data collection  

Agilent Xcalibur (Ruby, Gemini ultra) diffractometer
Absorption correction: multi-scan (*CrysAlis PRO*; Agilent, 2012 ▸) *T*_min_ = 0.770, *T*_max_ = 1
5767 measured reflections
3398 independent reflections
2814 reflections with *I* > 2σ(*I*)
*R*_int_ = 0.031

### Refinement  

*R*[*F* ^2^ > 2σ(*F* ^2^)] = 0.044
*wR*(*F* ^2^) = 0.109
*S* = 1.02
3398 reflections
222 parameters
6 restraints
H-atom parameters constrained
Δρ_max_ = 0.75 e Å^−3^
Δρ_min_ = −0.60 e Å^−3^

### 

Data collection: *CrysAlis PRO* (Agilent, 2012 ▸); cell refinement: *CrysAlis PRO*; data reduction: *CrysAlis PRO*; program(s) used to solve structure: *SIR2002* (Burla *et al.*, 2003 ▸); program(s) used to refine structure: *SHELXL97* (Sheldrick, 2008 ▸); molecular graphics: *ORTEP-3 for Windows* (Farrugia, 2012 ▸) and *Mercury* (Macrae *et al.*, 2006 ▸); software used to prepare material for publication: *SHELXL97*.

## Supplementary Material

Crystal structure: contains datablock(s) I. DOI: 10.1107/S2056989015023130/bq2402sup1.cif

Structure factors: contains datablock(s) I. DOI: 10.1107/S2056989015023130/bq2402Isup2.hkl

Supporting information file. DOI: 10.1107/S2056989015023130/bq2402Isup3.mol

Supporting information file. DOI: 10.1107/S2056989015023130/bq2402Isup4.cml

**Fig. 1.**
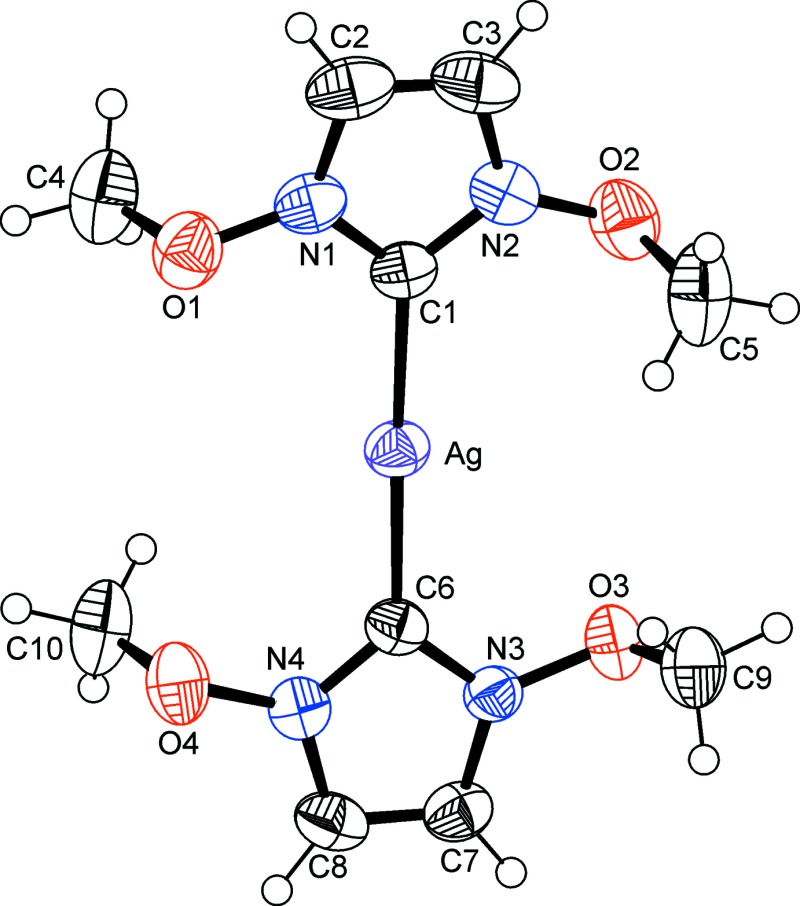
The molecular structure of the title compound, with atom labels and 50% probability displacement ellipsoids for non-H atoms. The hexafluoridophosphate ion is not shown.

. DOI: 10.1107/S2056989015023130/bq2402fig1.tif
The mol­ecular structure of the title compound, with atom labels and 50% probability displacement ellipsoids for non-H atoms. The hexa­fluorido­phosphate ion is not shown.

**Fig. 2.**
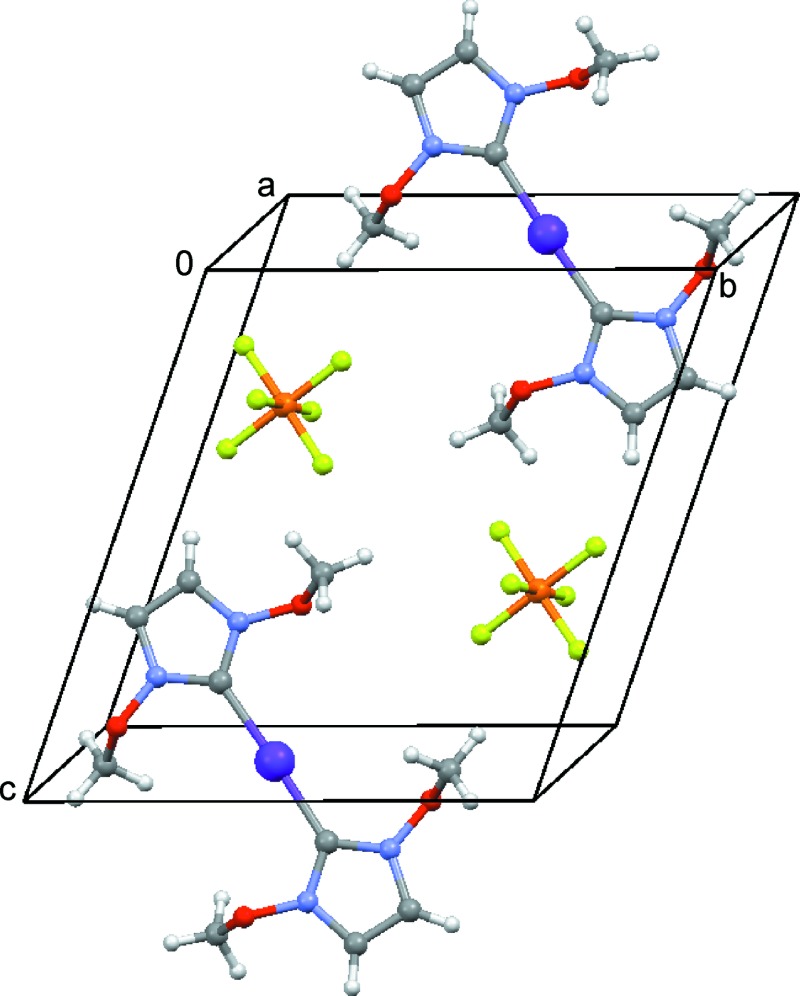
Unit cell of the title compound.

**Fig. 3.**
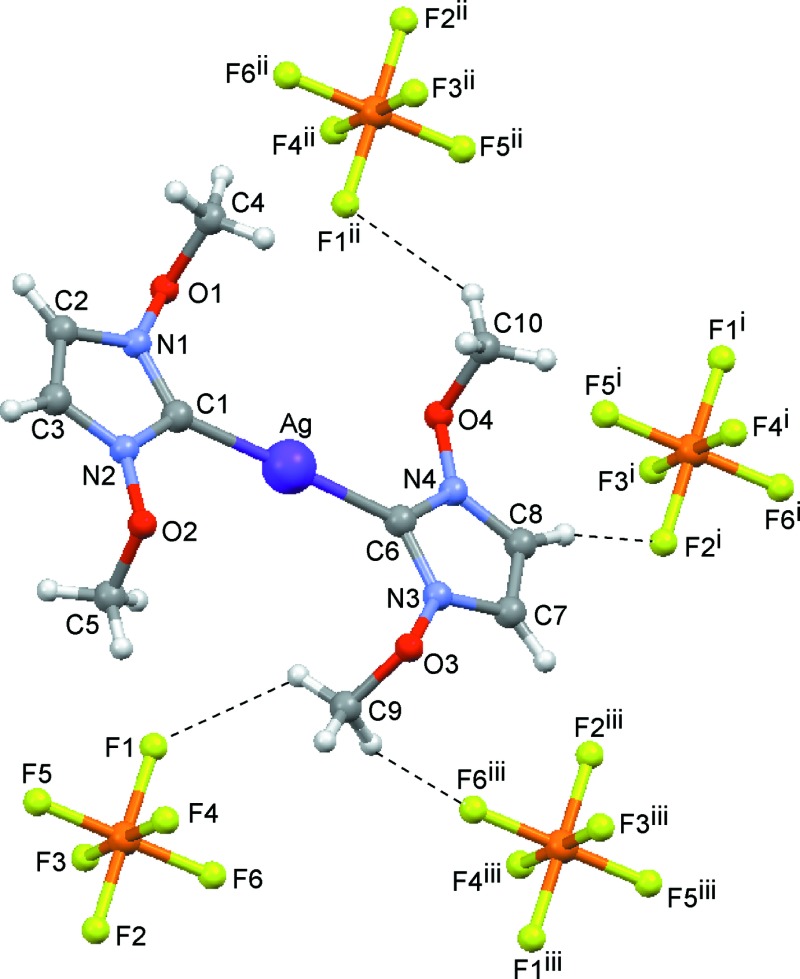
Interionic contacts in the crystal structure of the title compound. Symmetry codes: (i) x, 1 + y, z; (ii) 1 − x, 1 − y, 2 − z; (iii) 1 − x, 1 − y, 3 − z.

. DOI: 10.1107/S2056989015023130/bq2402fig2.tif
Unit cell of the title compound.

x y z x y z x y z . DOI: 10.1107/S2056989015023130/bq2402fig3.tif
Inter­ionic contacts in the crystal structure of the title compound. Symmetry codes: (i) *x*, 1 + *y*, *z*; (ii) 1 − *x*, 1 − *y*, 2 − *z*; (iii) 1 − *x*, 1 − *y*, 3 − *z*.

**Fig. 4.**
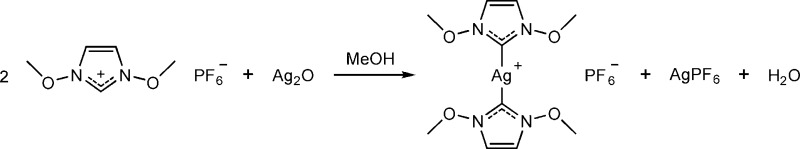
Reaction scheme.

. DOI: 10.1107/S2056989015023130/bq2402fig4.tif
Reaction scheme.

**Fig. 5.**
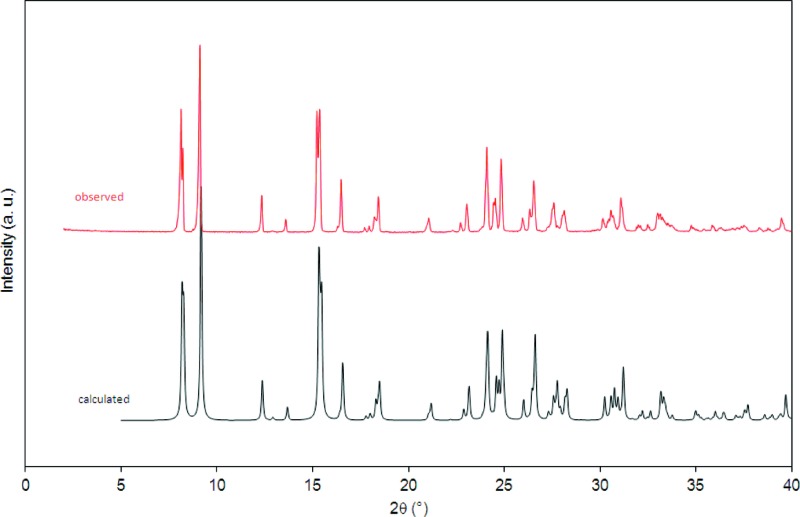
Observed and calculated powder X-ray diffraction data.

. DOI: 10.1107/S2056989015023130/bq2402fig5.tif
Observed and calculated powder X-ray diffraction data.

CCDC reference: 1439919

Additional supporting information:  crystallographic information; 3D view; checkCIF report

## Figures and Tables

**Table 1:** Hydrogen-bond geometry (Å, °)

*D*—H⋯*A*	*D*—H	H⋯*A*	*D*⋯*A*	*D*—H⋯*A*
C9—H9*A*⋯F1	0.97	2.57	3.193 (8)	122
C8—H8⋯F2^i^	0.94	2.46	3.382 (5)	165
C10—H10*B*⋯F1^ii^	0.97	2.54	3.334 (9)	140
C9—H9*C*⋯F6^iii^	0.97	2.58	3.516 (6)	163

Symmetry codes: (i) 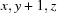; (ii) 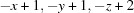; (iii) 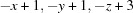.

## supplementary crystallographic information

### S1. Comment

N-Heterocyclic carbene (NHC)–silver complexes are valuable precursors for transmetalation to other metal NHC systems (Garrison & Youngs, 2005; Lin *et al.*, 2009; Lin & Vasam, 2007; Wang & Lin, 1998). In the crystal structure of the title compound, the central carbene–metal bonds C1—Ag and C6—Ag are 2.073 (4) and 2.070 (4) Å long, respectively, and deviate only slightly from linearity with an angle of 178.1 (2)°. The N—C—N 'carbene angles' are 101.0 (4)° and 100.4 (4)°, significantly smaller than the mean value of 104.5° in bis(NHC)–Ag complexes from the CSD (1002 values from 344 entries), but in line with related *N*-alkyloxy-substituted compounds (reference codes: DOJNIA and YUWZOG), where the angles range from 100.9° to 102.0° (Laus *et al.*, 2008 and 2010). The dihedral angle between the imidazole rings is 3.0 (3)°. The methoxy groups adopt *anti* conformation. The molecular structure is shown in Figure 1. The unit cell contains two ion pairs (Figure 2). The weakly coordinating hexafluorophosphate ion accepts several C—H···F hydrogen bonds (D'Oria & Novoa, 2008) from the cationic complex (Figure 3). The hydrogen bond geometries are summarized in Table 1.

### S2. Experimental

A suspension of 1,3-dimethoxyimidazolium hexafluorophosphate (1.0 g, 3.6 mmol) (Laus *et al.*, 2007) and Ag_2_O (0.40 g, 1.7 mmol) in MeOH (20 ml) was stirred at room temperature for 18 h (Figure 4), until the dark Ag_2_O was consumed. The desired product was filtered off (the filtrate contained the soluble AgPF_6_), washed with MeOH and Et_2_O and recrystallized from hot MeOH to yield colourless crystals (0.55 g, 62%). The PXRD (Cu *K*α radiation) of the bulk material was identical to the one calculated from the single-crystal diffraction data (Figure 5).

Melting point: 164–166 °C. ^1^H NMR (DMSO-d_6_, 300 MHz): δ 4.16 (s, 12H), 7.91 (s, 4H) p.p.m. ^13^C NMR (DMSO-d_6_, 75 MHz): δ 68.1 (4 C), 116.6 (4 C), 165.7 (2 C) p.p.m. IR (neat): ν 3172 (w), 3155 (w), 2951 (w), 1460 (w), 1440 (w), 1027 (*m*), 957 (*m*), 822 (*s*), 705 (*m*), 555 (*s*) cm^−1^.

### S3. Refinement

Carbon-bound H atoms were placed in calculated positions and refined riding on their respective carbon atom. Methyl H atoms were fitted to the experimental electron density by allowing them to rotate around the C—C bond with a fixed angle (AFIX 137). Isotropic displacement parameters were constrained with *U*_iso_(H) = 1.5*U*_eq_(C) for methyl H atoms and 1.2*U*_eq_(C) for other H atoms. The F atoms of the PF_6_ ions were restrained with a distance of P—F = 1.57 Å.

### Crystal data

**Table:**

[Ag(C_5_H_8_N_2_O_2_)_2_]PF_6_	*Z* = 2
*M**_r_* = 509.11	*F*(000) = 504
Triclinic, *P*1	*D*_x_ = 1.803 Mg m^−^^3^
Hall symbol: -P 1	Mo *K*α radiation, λ = 0.71073 Å
*a* = 7.5254 (7) Å	Cell parameters from 2189 reflections
*b* = 11.7221 (12) Å	θ = 3.1–28.5°
*c* = 11.8697 (12) Å	µ = 1.24 mm^−^^1^
α = 109.481 (9)°	*T* = 243 K
β = 100.698 (8)°	Prismatic fragment, colourless
γ = 100.052 (8)°	0.25 × 0.12 × 0.05 mm
*V* = 937.84 (17) Å^3^	

### Data collection

**Table:**

Agilent Xcalibur (Ruby, Gemini ultra) diffractometer	3398 independent reflections
Graphite monochromator	2814 reflections with *I* > 2σ(*I*)
Detector resolution: 10.3575 pixels mm^-1^	*R*_int_ = 0.031
ω scans	θ_max_ = 25.4°, θ_min_ = 3.2°
Absorption correction: multi-scan (*CrysAlis PRO*; Agilent, 2012)	*h* = −9→7
*T*_min_ = 0.770, *T*_max_ = 1	*k* = −12→14
5767 measured reflections	*l* = −14→14

### Refinement

**Table:**

Refinement on *F*^2^	Secondary atom site location: difference Fourier map
Least-squares matrix: full	Hydrogen site location: inferred from neighbouring sites
*R*[*F*^2^ > 2σ(*F*^2^)] = 0.044	H-atom parameters constrained
*wR*(*F*^2^) = 0.109	*w* = 1/[σ^2^(*F*_o_^2^) + (0.0489*P*)^2^ + 1.0584*P*] where *P* = (*F*_o_^2^ + 2*F*_c_^2^)/3
*S* = 1.02	(Δ/σ)_max_ < 0.001
3398 reflections	Δρ_max_ = 0.75 e Å^−^^3^
222 parameters	Δρ_min_ = −0.60 e Å^−^^3^
6 restraints	Extinction correction: *SHELXL97* (Sheldrick, 2008), Fc^*^=kFc[1+0.001xFc^2^λ^3^/sin(2θ)]^-1/4^
Primary atom site location: structure-invariant direct methods	Extinction coefficient: 0.0166 (15)

### Special details

**Table:**

Experimental. Absorption correction: *CrysAlis PRO*, Agilent Technologies, Version 1.171.36.20 (release 27–06-2012 CrysAlis171. NET) (compiled Jul 11 2012,15:38:31) Empirical absorption correction using spherical harmonics, implemented in SCALE3 ABSPACK scaling algorithm.
Geometry. All e.s.d.'s (except the e.s.d. in the dihedral angle between two l.s. planes) are estimated using the full covariance matrix. The cell e.s.d.'s are taken into account individually in the estimation of e.s.d.'s in distances, angles and torsion angles; correlations between e.s.d.'s in cell parameters are only used when they are defined by crystal symmetry. An approximate (isotropic) treatment of cell e.s.d.'s is used for estimating e.s.d.'s involving l.s. planes.
Refinement. Refinement of *F*^2^ against ALL reflections. The weighted *R*-factor *wR* and goodness of fit *S* are based on *F*^2^, conventional *R*-factors *R* are based on *F*, with *F* set to zero for negative *F*^2^. The threshold expression of *F*^2^ > σ(*F*^2^) is used only for calculating *R*-factors(gt) *etc*. and is not relevant to the choice of reflections for refinement. *R*-factors based on *F*^2^ are statistically about twice as large as those based on *F*, and *R*- factors based on ALL data will be even larger.

### Fractional atomic coordinates and isotropic or equivalent isotropic displacement parameters (Å^2^)

**Table:**

	*x*	*y*	*z*	*U*_iso_*/*U*_eq_	
Ag	0.23186 (5)	0.43947 (3)	0.95741 (3)	0.04281 (18)	
P	0.5262 (2)	0.18543 (11)	1.32827 (11)	0.0552 (4)	
F1	0.5321 (7)	0.2674 (3)	1.2468 (3)	0.0968 (10)	
F6	0.5226 (8)	0.3018 (3)	1.4400 (3)	0.1157 (13)	
F3	0.7426 (5)	0.2188 (6)	1.3712 (6)	0.1502 (16)	
F2	0.5166 (7)	0.1034 (3)	1.4097 (3)	0.0968 (10)	
F5	0.5222 (8)	0.0672 (3)	1.2160 (3)	0.1157 (13)	
F4	0.3055 (5)	0.1529 (6)	1.2871 (6)	0.1502 (16)	
O4	0.3885 (5)	0.7554 (3)	1.0537 (4)	0.0560 (9)	
O3	0.1139 (5)	0.4969 (3)	1.2341 (3)	0.0528 (9)	
O2	0.0715 (5)	0.1240 (3)	0.8695 (4)	0.0611 (10)	
C1	0.2102 (6)	0.2811 (4)	0.8048 (4)	0.0403 (10)	
O1	0.3470 (5)	0.3732 (4)	0.6798 (3)	0.0605 (10)	
N3	0.2052 (5)	0.6009 (3)	1.2185 (3)	0.0389 (8)	
N2	0.1483 (6)	0.1604 (4)	0.7865 (4)	0.0471 (10)	
N4	0.3151 (5)	0.7149 (3)	1.1359 (3)	0.0395 (9)	
N1	0.2605 (6)	0.2710 (4)	0.7000 (4)	0.0463 (9)	
C6	0.2497 (6)	0.5939 (4)	1.1125 (4)	0.0377 (10)	
C10	0.2488 (9)	0.7857 (6)	0.9781 (6)	0.0709 (17)	
H10A	0.1441	0.7126	0.9337	0.106*	
H10B	0.301	0.812	0.9192	0.106*	
H10C	0.2062	0.853	1.0298	0.106*	
C7	0.2416 (7)	0.7202 (4)	1.3035 (4)	0.0450 (11)	
H7	0.2208	0.7447	1.3828	0.054*	
C8	0.3132 (7)	0.7948 (4)	1.2498 (5)	0.0488 (12)	
H8	0.3533	0.8827	1.2831	0.059*	
C5	0.2106 (9)	0.1109 (7)	0.9606 (7)	0.0790 (19)	
H5A	0.2746	0.0506	0.9201	0.118*	
H5B	0.1516	0.0821	1.0157	0.118*	
H5C	0.3001	0.1912	1.008	0.118*	
C4	0.2153 (9)	0.4083 (6)	0.6024 (6)	0.0729 (17)	
H4A	0.1472	0.3355	0.5284	0.109*	
H4B	0.2814	0.472	0.579	0.109*	
H4C	0.1282	0.441	0.6471	0.109*	
C3	0.1572 (9)	0.0797 (5)	0.6757 (5)	0.0642 (15)	
H3	0.1188	−0.0081	0.645	0.077*	
C9	0.2456 (9)	0.4396 (5)	1.2843 (6)	0.0704 (17)	
H9A	0.3259	0.4172	1.2297	0.106*	
H9B	0.1786	0.3649	1.2917	0.106*	
H9C	0.3212	0.4981	1.3654	0.106*	
C2	0.2309 (8)	0.1495 (5)	0.6192 (5)	0.0630 (15)	
H2	0.2569	0.122	0.5415	0.076*	

### Atomic displacement parameters (Å^2^)

**Table:**

	*U*^11^	*U*^22^	*U*^33^	*U*^12^	*U*^13^	*U*^23^
Ag	0.0481 (3)	0.0401 (2)	0.0376 (2)	0.01478 (16)	0.01134 (16)	0.00972 (16)
P	0.0909 (12)	0.0415 (7)	0.0372 (7)	0.0268 (7)	0.0195 (7)	0.0134 (6)
F1	0.174 (3)	0.0702 (16)	0.0679 (16)	0.0446 (18)	0.0437 (18)	0.0397 (14)
F6	0.229 (4)	0.0686 (16)	0.0589 (16)	0.061 (2)	0.055 (2)	0.0154 (13)
F3	0.090 (3)	0.167 (4)	0.202 (5)	0.026 (2)	0.026 (3)	0.090 (4)
F2	0.174 (3)	0.0702 (16)	0.0679 (16)	0.0446 (18)	0.0437 (18)	0.0397 (14)
F5	0.229 (4)	0.0686 (16)	0.0589 (16)	0.061 (2)	0.055 (2)	0.0154 (13)
F4	0.090 (3)	0.167 (4)	0.202 (5)	0.026 (2)	0.026 (3)	0.090 (4)
O4	0.049 (2)	0.062 (2)	0.073 (2)	0.0183 (17)	0.0291 (19)	0.037 (2)
O3	0.054 (2)	0.0500 (19)	0.063 (2)	0.0109 (16)	0.0186 (18)	0.0318 (17)
O2	0.045 (2)	0.063 (2)	0.079 (3)	0.0088 (17)	0.019 (2)	0.033 (2)
C1	0.035 (2)	0.044 (3)	0.038 (3)	0.013 (2)	0.007 (2)	0.011 (2)
O1	0.052 (2)	0.072 (2)	0.062 (2)	0.0127 (19)	0.0166 (19)	0.032 (2)
N3	0.042 (2)	0.0373 (19)	0.035 (2)	0.0112 (16)	0.0067 (17)	0.0127 (16)
N2	0.041 (2)	0.045 (2)	0.053 (2)	0.0128 (18)	0.0122 (19)	0.0145 (19)
N4	0.032 (2)	0.044 (2)	0.045 (2)	0.0132 (17)	0.0110 (17)	0.0175 (18)
N1	0.044 (2)	0.051 (2)	0.041 (2)	0.0170 (19)	0.0116 (19)	0.0103 (18)
C6	0.030 (2)	0.038 (2)	0.041 (3)	0.0091 (18)	0.0049 (19)	0.0122 (19)
C10	0.073 (4)	0.092 (4)	0.079 (4)	0.030 (4)	0.035 (3)	0.059 (4)
C7	0.048 (3)	0.046 (3)	0.039 (3)	0.021 (2)	0.010 (2)	0.011 (2)
C8	0.046 (3)	0.037 (2)	0.053 (3)	0.011 (2)	0.004 (2)	0.008 (2)
C5	0.071 (4)	0.099 (5)	0.104 (5)	0.029 (4)	0.039 (4)	0.072 (4)
C4	0.072 (4)	0.091 (4)	0.082 (4)	0.030 (4)	0.028 (4)	0.055 (4)
C3	0.068 (4)	0.044 (3)	0.063 (4)	0.018 (3)	0.012 (3)	−0.001 (3)
C9	0.092 (5)	0.057 (3)	0.071 (4)	0.022 (3)	0.012 (3)	0.038 (3)
C2	0.062 (4)	0.067 (4)	0.047 (3)	0.025 (3)	0.012 (3)	0.003 (3)

### Geometric parameters (Å, º)

**Table:**

Ag—C6	2.070 (4)	N4—C6	1.332 (6)
Ag—C1	2.073 (4)	N4—C8	1.368 (6)
P—F3	1.550 (4)	N1—C2	1.377 (6)
P—F5	1.561 (3)	C10—H10A	0.97
P—F6	1.562 (3)	C10—H10B	0.97
P—F1	1.574 (3)	C10—H10C	0.97
P—F2	1.576 (3)	C7—C8	1.340 (7)
P—F4	1.580 (4)	C7—H7	0.94
O4—N4	1.379 (5)	C8—H8	0.94
O4—C10	1.424 (7)	C5—H5A	0.97
O3—N3	1.378 (5)	C5—H5B	0.97
O3—C9	1.438 (6)	C5—H5C	0.97
O2—N2	1.376 (5)	C4—H4A	0.97
O2—C5	1.426 (7)	C4—H4B	0.97
C1—N2	1.339 (6)	C4—H4C	0.97
C1—N1	1.341 (6)	C3—C2	1.331 (8)
O1—N1	1.375 (5)	C3—H3	0.94
O1—C4	1.433 (7)	C9—H9A	0.97
N3—C6	1.342 (6)	C9—H9B	0.97
N3—C7	1.368 (6)	C9—H9C	0.97
N2—C3	1.362 (7)	C2—H2	0.94
			
C6—Ag—C1	178.13 (17)	N3—C6—Ag	130.3 (3)
F3—P—F5	91.4 (3)	O4—C10—H10A	109.5
F3—P—F6	90.6 (4)	O4—C10—H10B	109.5
F5—P—F6	177.9 (3)	H10A—C10—H10B	109.5
F3—P—F1	91.7 (3)	O4—C10—H10C	109.5
F5—P—F1	91.0 (2)	H10A—C10—H10C	109.5
F6—P—F1	89.5 (2)	H10B—C10—H10C	109.5
F3—P—F2	89.3 (3)	C8—C7—N3	105.5 (4)
F5—P—F2	89.2 (2)	C8—C7—H7	127.2
F6—P—F2	90.2 (2)	N3—C7—H7	127.2
F1—P—F2	179.0 (3)	C7—C8—N4	104.8 (4)
F3—P—F4	178.9 (4)	C7—C8—H8	127.6
F5—P—F4	89.6 (3)	N4—C8—H8	127.6
F6—P—F4	88.4 (3)	O2—C5—H5A	109.5
F1—P—F4	88.8 (3)	O2—C5—H5B	109.5
F2—P—F4	90.3 (3)	H5A—C5—H5B	109.5
N4—O4—C10	110.1 (4)	O2—C5—H5C	109.5
N3—O3—C9	110.8 (4)	H5A—C5—H5C	109.5
N2—O2—C5	111.5 (4)	H5B—C5—H5C	109.5
N2—C1—N1	101.0 (4)	O1—C4—H4A	109.5
N2—C1—Ag	129.2 (3)	O1—C4—H4B	109.5
N1—C1—Ag	129.8 (3)	H4A—C4—H4B	109.5
N1—O1—C4	110.7 (4)	O1—C4—H4C	109.5
C6—N3—C7	114.2 (4)	H4A—C4—H4C	109.5
C6—N3—O3	122.1 (4)	H4B—C4—H4C	109.5
C7—N3—O3	123.4 (4)	C2—C3—N2	106.6 (5)
C1—N2—C3	113.7 (4)	C2—C3—H3	126.7
C1—N2—O2	122.1 (4)	N2—C3—H3	126.7
C3—N2—O2	124.1 (4)	O3—C9—H9A	109.5
C6—N4—C8	115.0 (4)	O3—C9—H9B	109.5
C6—N4—O4	122.1 (4)	H9A—C9—H9B	109.5
C8—N4—O4	122.8 (4)	O3—C9—H9C	109.5
C1—N1—O1	122.5 (4)	H9A—C9—H9C	109.5
C1—N1—C2	114.1 (5)	H9B—C9—H9C	109.5
O1—N1—C2	123.2 (4)	C3—C2—N1	104.6 (5)
N4—C6—N3	100.5 (4)	C3—C2—H2	127.7
N4—C6—Ag	129.2 (3)	N1—C2—H2	127.7

### Hydrogen-bond geometry (Å, º)

**Table:**

*D*—H···*A*	*D*—H	H···*A*	*D*···*A*	*D*—H···*A*
C9—H9*A*···F1	0.97	2.57	3.193 (8)	122
C8—H8···F2^i^	0.94	2.46	3.382 (5)	165
C10—H10*B*···F1^ii^	0.97	2.54	3.334 (9)	140
C9—H9*C*···F6^iii^	0.97	2.58	3.516 (6)	163

Symmetry codes: (i) *x*, *y*+1, *z*; (ii) −*x*+1, −*y*+1, −*z*+2; (iii) −*x*+1, −*y*+1, −*z*+3.
