# Supplementary figures and images for: Crystal structure of bis­(1,3-di­meth­oxy­imidazolin-2-yl­idene)silver(I) hexa­fluorido­phosphate, N-heterocyclic carbene (NHC) complex
Source: Acta Crystallogr E Crystallogr Commun. 2015 Dec 9;71(Pt 12):m251–2. doi: 10.1107/S2056989015023130 (PMC4719859; doi:10.1107/S2056989015023130)

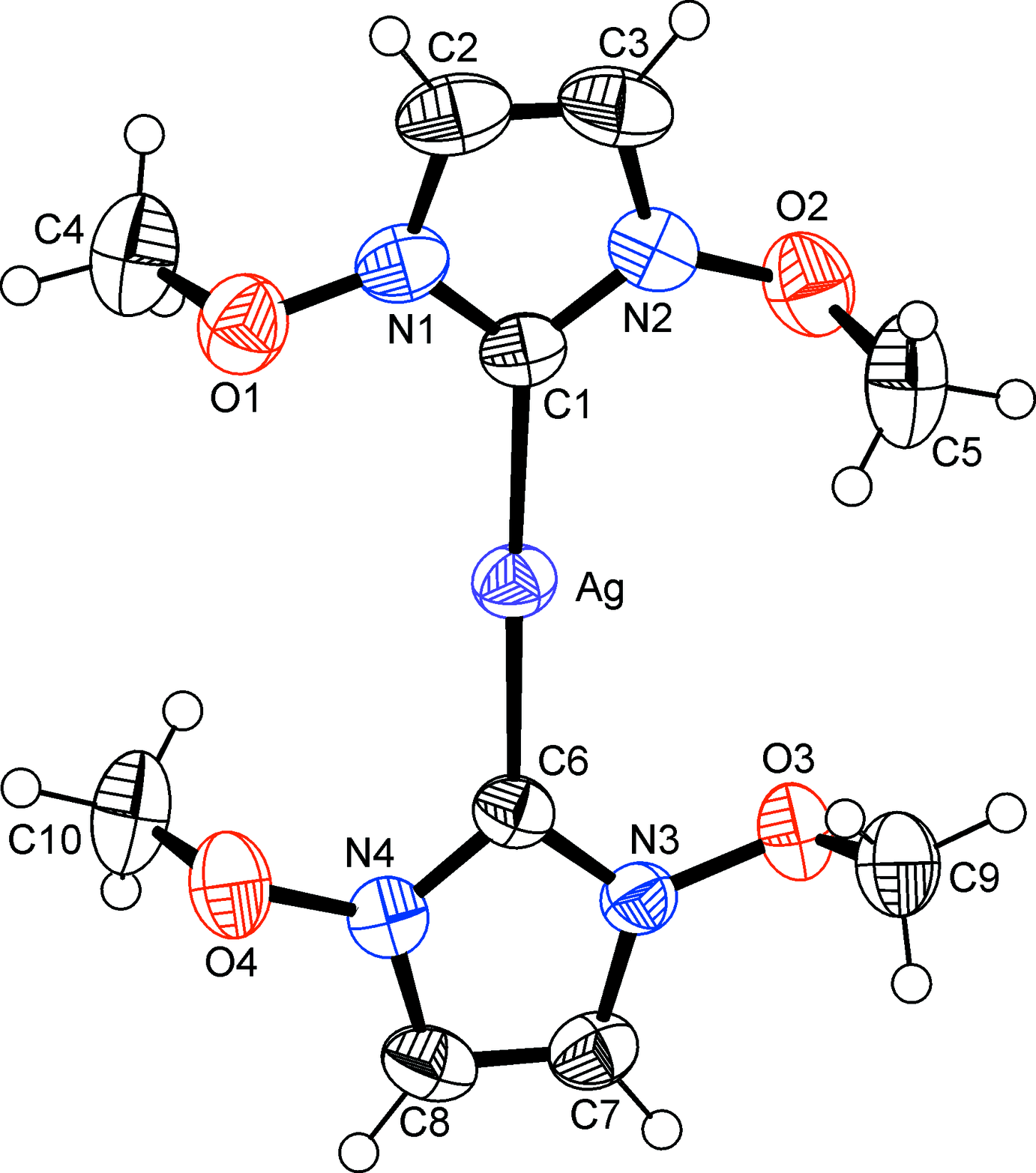

Supplement: Supplementary file 5 [file e-71-0m251-fig1.tif]

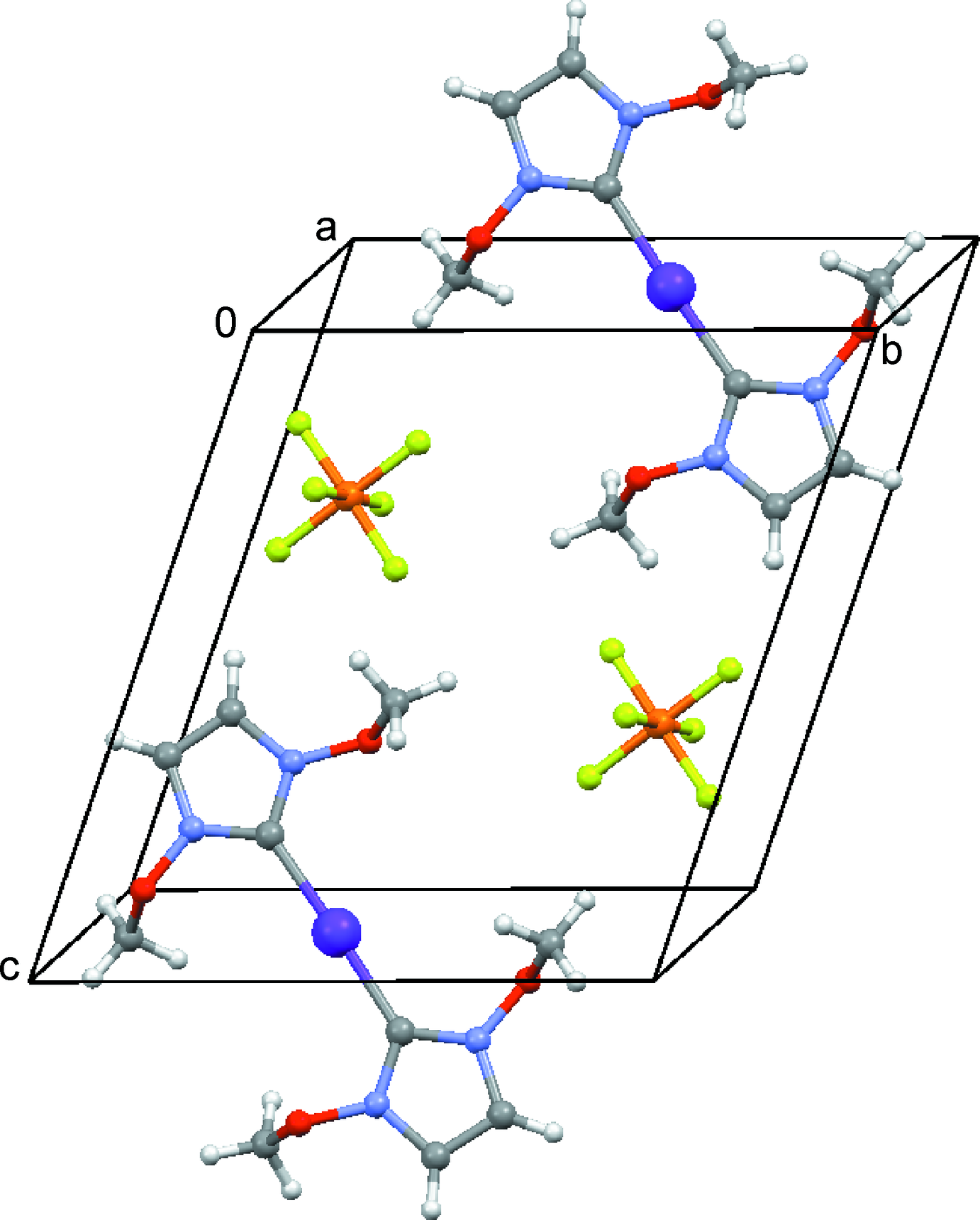

Supplement: Supplementary file 6 [file e-71-0m251-fig2.tif]

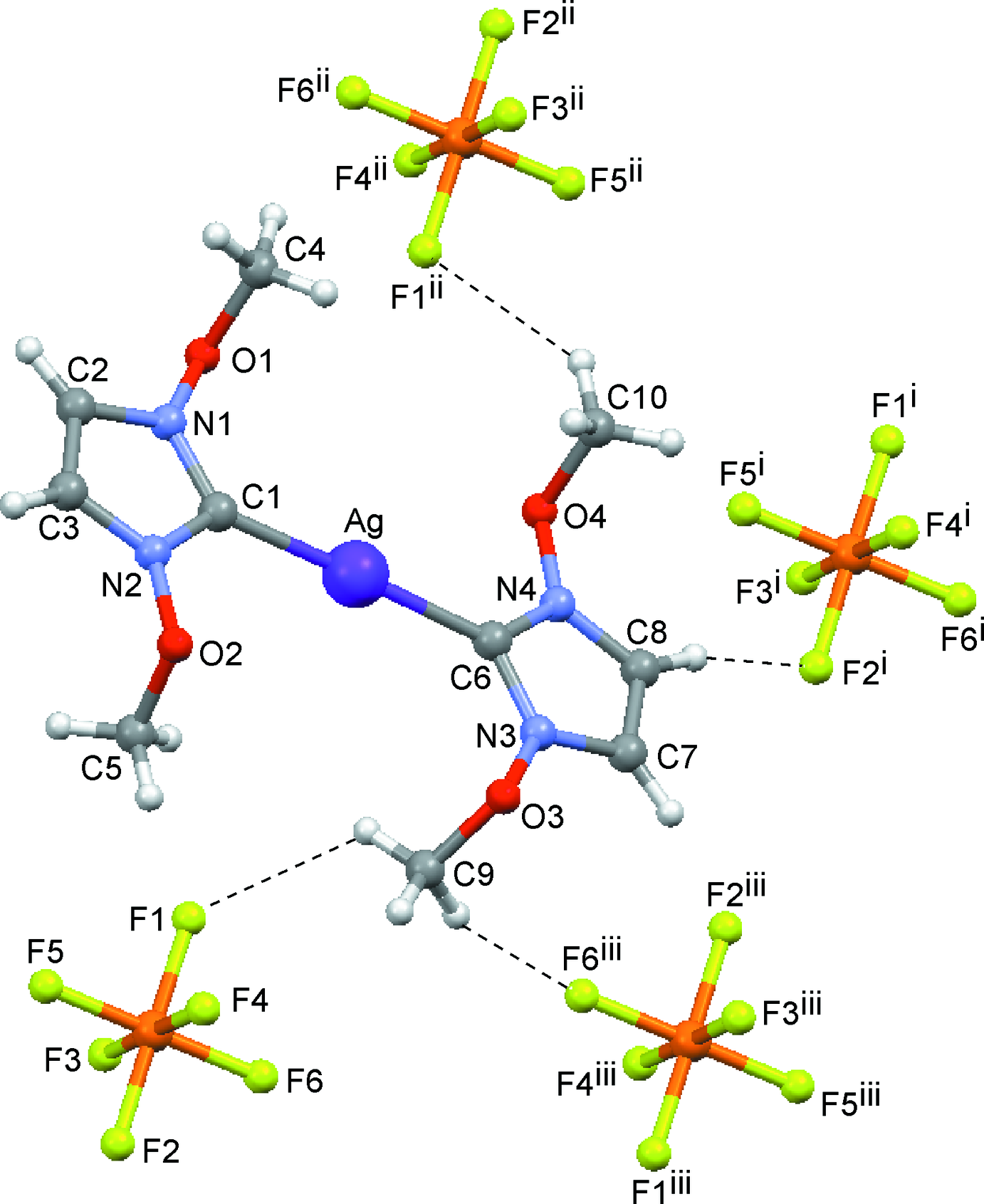

Supplement: Supplementary file 7 [file e-71-0m251-fig3.tif]

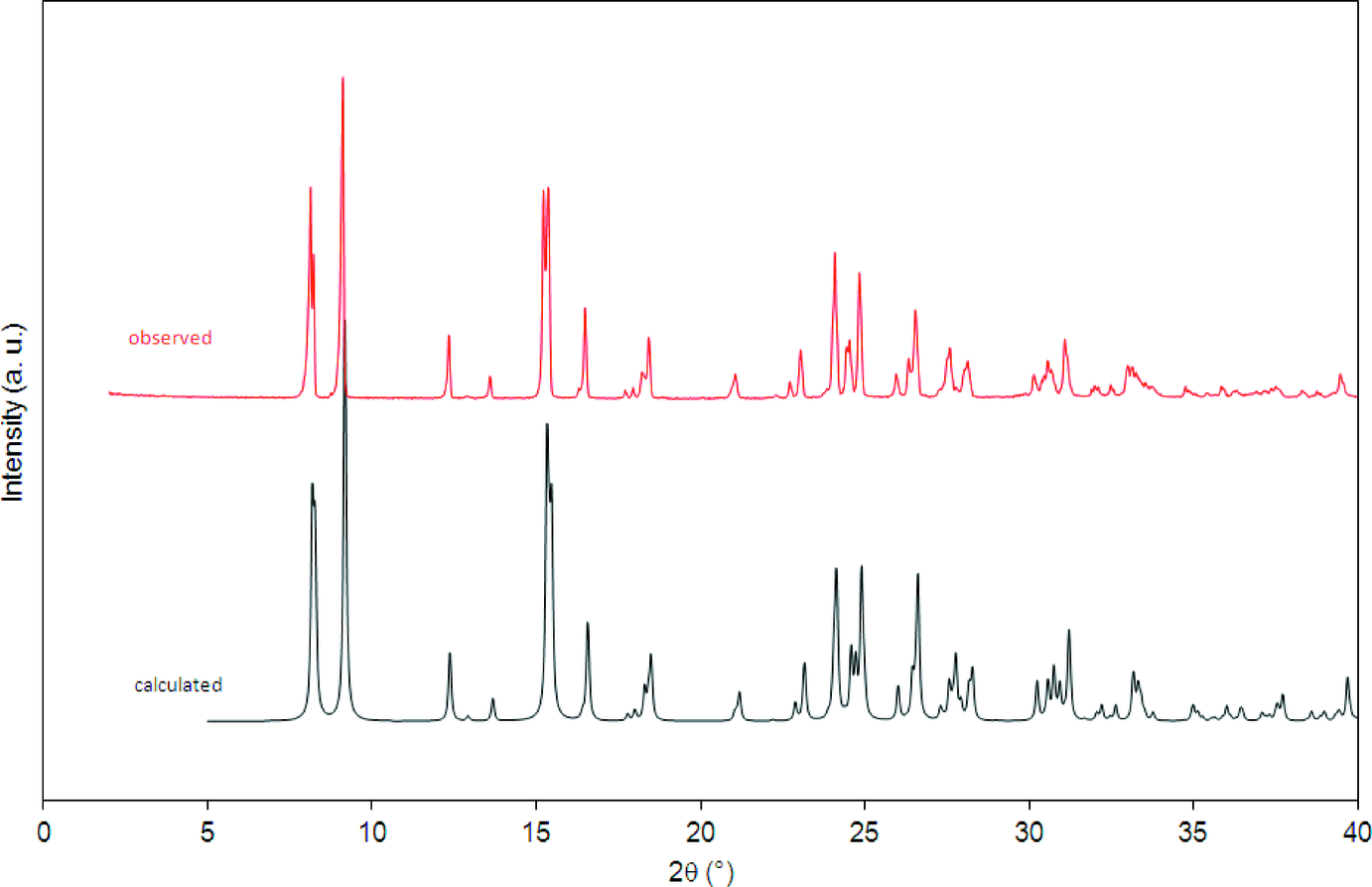

Supplement: Supplementary file 9 [file e-71-0m251-fig5.tif]
